# Fluorine-19 Magnetic Resonance Imaging and Positron Emission Tomography of Tumor-Associated Macrophages and Tumor Metabolism

**DOI:** 10.1155/2017/4896310

**Published:** 2017-12-05

**Authors:** Soo Hyun Shin, Sang Hyun Park, Se Hun Kang, Seung Won Kim, Minsun Kim, Daehong Kim

**Affiliations:** ^1^Molecular Imaging Branch, Division of Convergence Technology, National Cancer Center, Ilsanro-ro 323, Ilsandong-gu, Goyang 10408, Republic of Korea; ^2^Animal Molecular Imaging Unit, Research Institute, National Cancer Center, Ilsanro-ro 323, Ilsandong-gu, Goyang 10408, Republic of Korea

## Abstract

The presence of tumor-associated macrophages (TAMs) is significantly associated with poor prognosis of tumors. Currently, magnetic resonance imaging- (MRI-) based TAM imaging methods that use nanoparticles such as superparamagnetic iron oxide and perfluorocarbon nanoemulsions are available for quantitative monitoring of TAM burden in tumors. However, whether MRI-based measurements of TAMs can be used as prognostic markers has not been evaluated yet. In this study, we used positron emission tomography (PET) with ^18^F-2-fluoro-2-deoxy-D-glucose (^18^F-FDG) as a radioactive tracer and fluorine-19- (^19^F-) MRI for imaging mouse breast cancer models to determine any association between TAM infiltration and tumor metabolism. Perfluorocarbon nanoemulsions were intravenously administered to track and quantify TAM infiltration using a 7T MR scanner. To analyze glucose uptake in tumors,^ 18^F-FDG-PET images were acquired immediately after ^19^F-MRI. Coregistered ^18^F-FDG-PET and ^19^F-MR images enabled comparison of spatial patterns of glucose uptake and TAM distribution in tumors. ^19^F-MR signal intensities from tumors exhibited a strong inverse correlation with ^18^F-FDG uptake while having a significant positive correlation with tumor growth from days 2 to 7. These results show that combination of ^19^F-MRI and ^18^F-FDG-PET can improve our understanding of the relationship between TAM and tumor microenvironment.

## 1. Introduction

Many types of tumors with poor prognosis are characterized by dense infiltration of tumor-associated macrophages (TAMs) [1–3]. Crosstalk between TAMs and tumor cells through anti-inflammatory cytokines such as interleukin-10 contributes to various aspects of tumor progression by activities such as promoting tumor angiogenesis [4–6], supporting destruction of basal extracellular matrix [7, 8], and facilitating metastasis [9]. Thus, TAMs have been drawing attention as key diagnostic, prognostic, and therapeutic targets for characterization and treatment of tumors [10–12].

Several imaging methods have been developed for noninvasive analysis of distribution and quantification of TAMs in tumors. One of these methods is the nanoparticle-based magnetic resonance imaging (MRI) cell-tracking method, which exploits the high phagocytic activity of macrophages to passively label them with nanoparticles through intravenous administration. Superparamagnetic iron oxide (SPIO) nanoparticles and perfluorocarbon (PFC) nanoemulsions are widely used as TAM-labeling contrast agents. With SPIO nanoparticles, TAMs are visualized as hypointense spots on T2-weighted MR images. These nanoparticles have a high potential for clinical translation owing to their approval by the Food and Drug Administration (e.g., Feraheme) [13, 14]. Upon fluorination with PFC nanoemulsions, TAMs are visualized as “hot spots” by fluorine-19- (^19^F-) MRI [15–18]. Because of the lack of ^19^F atoms in biological tissues, ^19^F-MRI confirms the presence of TAMs once ^19^F signals are detected; it also enables a simple quantification process, because the number of ^19^F spins is linearly correlated with the corresponding MR signal intensity.

While both SPIO and PFC have been shown to be effective for monitoring and quantifying TAMs, whether TAM burden quantified through these nanoparticle-based methods is associated with tumor development has yet to be examined. To investigate the prognostic implications of MRI-based TAM monitoring, another imaging modality designed for observing tumor behavior may be concurrently used, along with histopathological analysis. We hypothesized that positron emission tomography (PET) with ^18^F-2-fluoro-2-deoxy-D-glucose (^18^F-FDG) as a radioactive tracer can serve such a purpose, since it is widely practiced as a clinical routine for staging tumor malignancy through measurement of tumor glucose uptake [19]. Simultaneous MRI tracking of TAMs and ^18^F-FDG-PET imaging of tumor metabolism might help determine the prognostic potential of MRI-based TAM tracking and provide new insights to understanding tumor physiology.

Here, we report the preliminary results from combining ^19^F-MRI and ^18^F-FDG-PET for monitoring TAM infiltration and tumor metabolism. The feasibility of spatial correlation of TAM distribution and glucose metabolism patterns was investigated, and significant correlations were observed between ^19^F-MR signal intensity and PET parameters. Overall, we demonstrate that combination of ^19^F-MR-based TAM tracking and ^18^F-FDG-PET imaging could provide opportunities for noninvasive yet precise profiling of tumor microenvironment and behavior.

## 2. Materials and Methods

### 2.1. Preparation of PFC Emulsion

PFC nanoemulsions were synthesized in accordance with a previous protocol, with a minor modification [20]. Lutrol F68 (60 mg/mL; BASF, Ludwigshafen, Germany) was dissolved in phosphate buffered saline (PBS; Gibco, Waltham, MA). Perfluoro-15-crown-5-ether (60% w/v; Oakwood Chemicals, Estill, SC) was thoroughly mixed into this solution using a micromixer. The mixture was emulsified by sonication in ice-cold water in a pulsed mode (2 s run and 2 s off) at 1.5 W for 10 min using a sonicator (Sonicator 3000; Misonix, Farmingdale, NY). In the last 2-3 cycles of sonication, 1,1′-dioctadecyl-3,3,3′3′-tetramethylindocarbocyanine perchlorate (DiI; 4 *μ*l/mL; Molecular Probes, Eugene, OR) was added for fluorescence. The resultant emulsions were then filter-sterilized through 0.45 and 0.2 *μ*m filters (Sartorius Stedim, Aubagne, France) and stored at 4°C until use. The size and polydispersity index of the PFC nanoemulsions were determined to be 160 nm and 0.08, respectively, using dynamic light scattering (Malvern Zetasizer, Worcestershire, UK).

### 2.2. Animal Models

All animal experiments were performed in accordance with the guidelines of the Institutional Animal Care and Use Committee (approval number, NCC-15-249) of the National Cancer Center, Korea. 4T1 mouse breast cancer cells (American Type Culture Collection, Manassas, VA) were cultured in Roswell Park Memorial Institute-1640 medium (Hyclone, Logan, UT) supplemented with 10% fetal bovine serum (Cellgro, Tewksbury, MA) and 1% antibiotic solution (Gibco) at 37°C in a 5%-CO_2_ incubator. For establishing a 4T1-tumor model, 5 × 10^5^ cells were suspended in 100 *μ*l of 5 mg/mL Matrigel (Corning, Corning, NY) and subcutaneously injected into the left and right flanks of 6-week-old female Balb/c mice (Japan SLC, Hamamatsu, Japan). Tumors (*n* = 16) were grown until they reached a size of 50–100 mm^3^. The mice were intravenously given 200 *μ*l of the PFC nanoemulsions 48 h before the first MRI and ^18^F-FDG-PET scan.

### 2.3. *In Vivo* MRI

MR images were acquired using a 7T scanner (BioSpec 70/20 USR; Bruker, Billerica MA) and a custom-made ^1^H/^19^F-double-tune 35 mm volume coil. A custom-built animal bed was used for transferring mice to the PET scanner without altering their posture. The mice were sedated with 2% isoflurane in 100% oxygen, and their respiration rates were monitored during imaging. Anatomical proton MR images were acquired using the rapid acquisition with relaxation enhancement (RARE) sequence. T2-weighted images were acquired with the following parameters: repetition time (TR), 2600 ms; echo time (TE), 30 ms; slice thickness (ST), 1 mm; RARE factor, 4; number of acquisitions (NA), 2; matrix size, 256 × 192; and field of view (FOV), 3.5 × 2.5 cm. For ^19^F image acquisition, the fast low angle shot sequence was used with the following parameters: TR, 100 ms; TE, 2.5 ms; ST, 2 mm; NA, 256; receiver bandwidth, 25 kHz; FOV, 3.5 cm × 2.5 cm; and matrix size, 64 × 48. A reference tube containing 6 mg/mL PFC nanoemulsions entrapped in acrylamide gel was placed next to the mice for tumor signal normalization. The mice were imaged 2 (day 2) and 7 (day 7) days after administration of the PFC nanoemulsions. ^19^F-MR images were acquired only on day 2.

### 2.4. PET/CT and Image Analysis

In order to maintain the orientation of the mice, PET/CT images were acquired immediately after MRI. The mice were fasted for 6 h before PET/CT. They were anesthetized with 2% isoflurane in 100% oxygen. Body temperature was maintained throughout the imaging procedure using a heating lamp and pad. ^18^F-FDG was prepared by an automated module (NEPTIS® Nx3 system, ORA, Philippeville, Belgium) using fluoride-18 generated by our on-site cyclotron (RDS-111, Siemens, Munich, Germany). The mice were intravenously injected with 18.5 MBq of ^18^F-FDG 40 min before PET. PET-CT fusion images were acquired through a three-dimensional acquisition mode (eXplore VistaCT, GE, Fairfield, CT) using the following X-ray parameters for CT: 250 *μ*A tube current and 40 kV voltage for 6 min; resolution, 200 *μ*m; and number of projections, 360. For PET images, the mice passed through the 6 cm diameter × 4.6 cm deep FOV of PET detector, and the voxel size of the reconstructed images was 0.3875 × 0.3875 × 0.775 mm. The images were acquired for 9 minutes per bed position and reconstructed by iterative reconstruction using the two-dimensional ordered subset expectation maximization method (32 subsets, 2 iterations). Normalization and scatter and attenuation correction were also applied for PET images. The images were normalized to standardized uptake values (SUVs) using the following formula: SUV = decay-corrected mean tissue activity concentration (Bq/ml)/[injected dose (Bq)/body weight (g)]. SUV_max_ was measured as the maximum SUV in a given region of interest. Percentage injected dose per gram tissue (% ID/g) was calculated as follows: [mean tumor activity concentration (Bq/ml)/(injected dose (Bq) × density of a tumor (g/ml))] × 100%. All image analyses were performed using the OsiriX imaging software (Pixmeo SARL, Bernex, Switzerland).

### 2.5. Histological Examination and Immunofluorescence Staining

The mice were euthanized upon completion of imaging experiments. Tumors were excised, fixed in 4% paraformaldehyde (Sigma Aldrich, St. Louis, MO) for 24 h, embedded in paraffin blocks, and cut into 4 *μ*m thick sections for hematoxylin and eosin (H&E) staining. The H&E-stained sections were imaged using the Aperio Scan Scope XT system (Leica Biosystems, Heidelerg, Germany) at 200x magnification.

For immunofluorescence staining, the fixed tumors were frozen in the optimal cutting temperature compound at −80°C and cut into 6 *μ*m thick sections using a Cryotome. Fluorescein isothiocyanate- (FITC-) conjugated rat anti-mouse F4/80 antibody (AbCam, Cambridge, MA) was used for staining macrophages. The stained sections were observed with a fluorescence microscope (Axio Obsever.Z1, Zeiss, Germany).

### 2.6. Statistical Analysis

All statistical data were analyzed using GraphPad Prism 5 (GraphPad Software, La Jolla, CA). Correlations among ^19^F-MRI signal intensities, tumor volume, and PET parameters (including SUV, SUV_max_, and percentage injected dose per gram tissue [% ID/g]) were analyzed by Pearson's correlation coefficient analysis. Absolute correlation coefficients ≥ 0.5 were considered as indicating strong correlation [21]. A *p* value < 0.05 was considered to be statistically significant.

## 3. Results and Discussion

**Figure 1:**
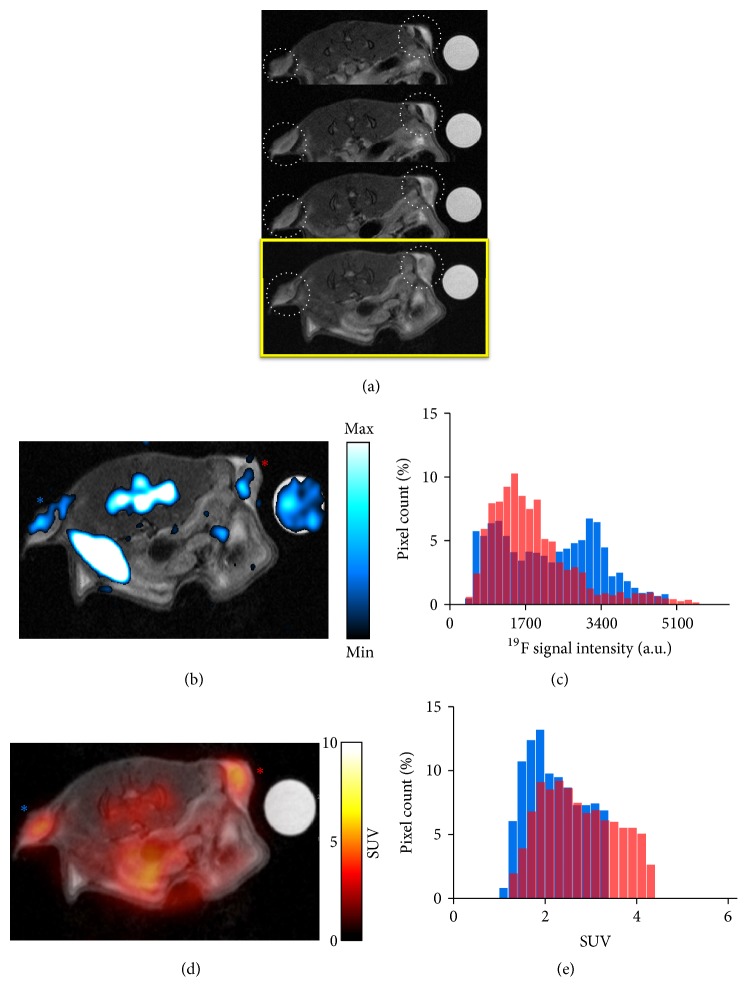
*In vivo*  ^19^F-MRI and PET and histogram analysis on day 2. (a) Serial ^1^H MR image slices with tumors indicated with dotted white circles. The last slice in yellow box is the slice that is coregistered with corresponding ^19^F-MR image and ^18^F-FDG-PET image in (b) and (d), respectively. (b) Superimposition of an anatomical proton MR image and its corresponding ^19^F-MR image. ^19^F signals are detected not only from tumors, but also from the bone marrow, the spleen, and a reference tube placed on the right side of the mouse. (c) Histogram of ^19^F pixel intensities from left and right tumors. Blue and red are from left (blue asterisk) and right (red asterisk) tumors in (b). (d) Coregistration of the same anatomical MR image as that used in image A and its corresponding ^18^F-FDG-PET image. (e) Histogram of PET SUV from left and right tumors. Blue and red are from left (blue asterisk) and right (red asterisk) tumors in (d). ^19^F, fluorine-19; MRI, magnetic resonance imaging; PET, positron emission tomography; 2-FDG, fluoro-2-deoxy-D-glucose; SUV, standardized uptake value.

^19^F-MRI and ^18^F-FDG-PET images were first acquired 2 days after administration of PFC nanoemulsions (Figure 1). ^19^F-MR signals were detected not only from tumors but also from naturally macrophage-rich tissues such as the spleen and bone marrow (Figure 1(b)). Coregistration of proton MR images and their corresponding PET images demonstrated tumor glucose metabolism in the same slices in which TAMs were visualized in ^19^F-MR images (Figure 1(d)). While ^18^F-FDG-PET signals appeared to be homogeneously distributed in tumors, ^19^F signals exhibited relatively heterogeneous intratumoral distribution, with higher signals emanating from the periphery of tumors. This difference in spatial distribution was further highlighted by the histogram findings, which revealed different frequency distributions of ^19^F signals and consistent SUV distribution between the left and right tumors (Figures 1(c) and 1(e)).

**Figure 2:**
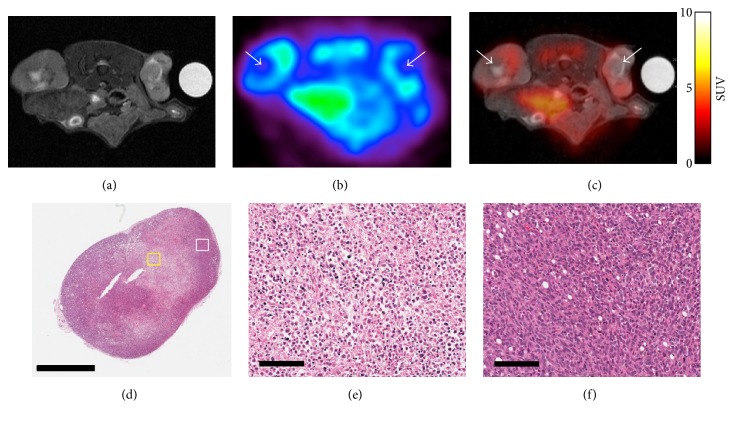
PET-MRI and histological findings on day 7. (a) Axial MR image of a mouse and (b) the corresponding PET image were merged. (c) The coregistered MR-PET image shows overlapping of low SUV regions and high MR signal regions in tumors (white arrow in (b) and (c)). (d) Overview of an H&E-stained section of a tumor excised after PET-MRI (scale bar: 2 mm). (e) High-magnification view of the central region of the H&E-stained section (indicated by a yellow box in (d); scale bar: 100 *μ*m); fragmented nuclei and disrupted cell morphology are observed, along with hypocellularity. (f) High-magnification view of the peripheral region of the tumor (white box in (d); scale bar: 100 *μ*m); cell morphology and nuclei are intact, while the cells are densely packed. PET, positron emission tomography; MRI, magnetic resonance imaging; SUV, standardized uptake value; H&E, hematoxylin and eosin.

Proton MRI and ^18^F-FDG-PET were repeated on day 7, and the imaging data were coregistered (Figure 2). In PET images, SUV hypointensities were observed at the centers of tumors, which corresponded to hyperintense signals observed in T2-weighted MR images (Figures 2(b) and 2(c)). Considering a previous study that reported an association between necrosis and high-intensity signals on T2-weighted MR images [22], it is likely that the colocalization of low SUVs and hyperintense T2-weighted MR signals observed in the present study represents the formation a necrotic core. This possibility was further supported by the histological findings, which revealed hypocellularity at the center of tumor sections and intact cell morphology at the periphery (Figures 2(d)–2(f)).

**Figure 3:**
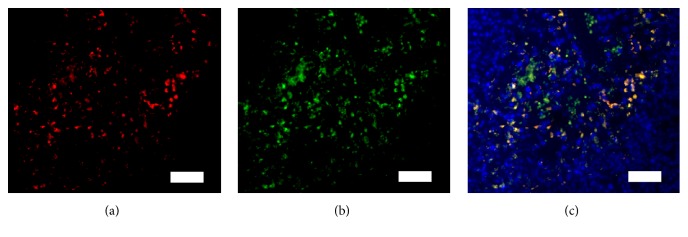
Immunofluorescence staining of TAMs reveals colocalization of TAMs and PFC nanoemulsions (scale bar: 50 *μ*m). (a) DiI on PFC nanoemulsions. (b) FITC on F4/80 antibodies. (c) Merged images of DAPI, DiI, and FITC staining showing colocalization of TAMs and PFC nanoemulsions (yellow); TAMs that are not labeled with PFC nanoemulsions are also observed (green). TAM, tumor-associated macrophage; PFC, perfluorocarbon; DiI, 1,1′-dioctadecyl-3,3,3′3′-tetramethylindocarbocyanine perchlorate; FITC, fluorescein isothiocyanate; DAPI, 4′,6-diamidino-2-phenylindole.

The findings of immunofluorescence staining—performed to confirm that the ^19^F-MR signals represent TAMs—demonstrated the colocalization of DiI with PFC nanoemulsions and F4/80-positive cells (Figure 3). While significant proportions of TAMs were labeled with PFC nanoemulsions, unlabeled TAMs were also detected (Figure 3(c)). This partial labeling of TAMs is consistent with the findings of previous studies [17, 23]. Further studies are needed to identify methods for achieving full saturation of endogenous macrophages with PFC nanoemulsions for more accurate quantification of TAMs.

**Figure 4:**
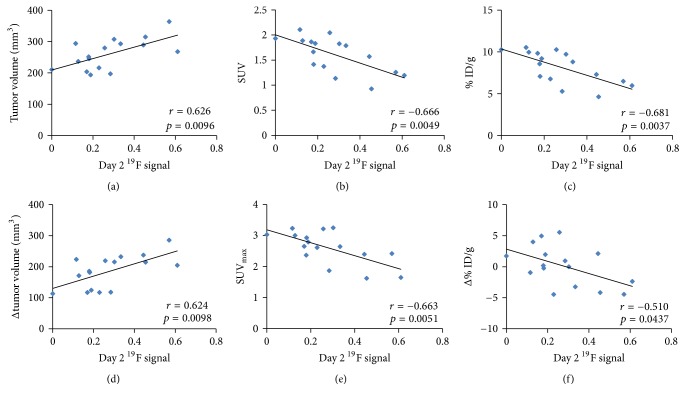
Correlation of ^19^F-MR signal intensity with tumor growth and PET parameters. (a) Tumor volume, (b) SUV, and (c) % ID/g measured on day 7 as functions of ^19^F-MR signal intensities measured on day 2. (d) Change in tumor growth from day 2 to day 7, (e) SUV_max_ measured on day 7, and (f) change in % ID/g from day 2 to day 7 as functions of ^19^F-MR signal intensities measured on day 2. ^19^F, fluorine-19; MR, magnetic resonance; PET, positron emission tomography; SUV, standardized uptake value; % ID/g, percentage injected dose per gram tissue; SUV_max_, maximum SUV.

^19^F-MR signal intensities from tumors measured on day 2 were correlated with tumor volume and various PET parameters (Table 1). Tumor ^19^F-MR signal intensities measured on day 2 exhibited significantly strong correlations with tumor volume measured on day 7 (*r* = 0.626; *p* < 0.01) and tumor growth between days 2 and 7 (*r* = 0.624; *p* < 0.01; Figures 4(a) and 4(d)). All PET parameters measured on day 7 exhibited strong negative correlations with ^19^F-MR signal intensities measured on day 2 (SUV: *r* = −0.666, *p* < 0.005; % ID/g: *r* = −0.681, *p* < 0.005; and SUV_max_: *r* = −0.663, *p* < 0.01; Figures 4(b), 4(c), and 4(e)). Changes in PET parameters over time were also correlated with ^19^F-MR signal intensity in a similar manner as the measurements on day 7—changes in SUV (*r* = −0.519, *p* < 0.05) and % ID/g (*r* = −0.510, *p* < 0.05) exhibited strong negative correlations (Figure 4(f)) with ^19^F-MR signal intensity. There was no correlation between ^19^F-MR signal intensity and any of the measurements recorded on day 2 or change in SUV_max_ (|*r*| < 0.2, *p* > 0.5).

Considering the protumoral activities of TAMs, we initially hypothesized that ^19^F-MR signal intensity would be positively correlated with all of the parameters evaluated in the present study. While the positive correlation of ^19^F-MR signal intensity with tumor volume and growth can be explained by the effects of TAMs on tumor development, the negative correlation between ^19^F-MR signal intensity and PET measurements recorded on day 7, which often serve as indicators of tumor malignancy, seem to be paradoxical. A possible explanation for this inverse correlation is the decrease in average tumor metabolism due to the formation of the necrotic core, which was observed in PET/MR images acquired on day 7 as well as in histological specimens (Figure 2). It is widely known that aggressive tumors often outgrow their blood supply, upon which their central regions are exposed to chronic ischemia, which ultimately leads to necrosis. In breast cancer, formation of such necrotic cores is associated with an accelerated clinical course and poor prognosis [24, 25]. Similarly, the formation of necrotic zones in breast tumor models used in this study—visualized as decreased ^18^F-FDG uptake at the center of tumors—might represent the aggressiveness and malignancy of tumors. To investigate this possibility, tumor models with the same genetic background but different degrees of malignancies should be evaluated by imaging along with rigorous histopathological analysis.

The lack of correlation between ^19^F-MR signal intensity and PET parameters measured on day 2 should also be noted. A previous histopathological study had reported that immune-cell infiltration and ^18^F-FDG-PET SUV are not significantly correlated [26]. Nevertheless, there is a possibility that varying the timing of monitoring will generate different results. Using both SPIO and PFC labeling, Makela et al. showed that distribution of TAMs varies significantly on the basis of tumor size at the time of monitoring [17]. Future studies should evaluate whether the characteristics of TAMs, too, change along with their intratumoral distribution over time. It should also be determined if any correlation exists between ^19^F-MR signal intensity and PET parameters concurrently measured at a later time point in tumor growth.

The combination of proton MRI and PET has been widely studied, with the aim of gaining a comprehensive understanding of tumor physiology and differentiating tumor subtypes by monitoring various aspects of the tumor microenvironment [27]. Several MR parametric methods, such as chemical exchange saturation transfer imaging, dynamic contrast enhanced MRI, and apparent diffusion coefficient mapping, have been used for measuring tumor acidosis, perfusion, and necrosis [28–30]. In terms of ^18^F-FDG-PET, a myriad of analytic approaches, including texture analysis, are being studied for better assessment of glucose metabolism patterns and enhanced characterization of tumors [31]. Considering these developments in both MRI and PET approaches for tumor characterization, it is envisioned that our knowledge of tumor microenvironment would be further enriched through the combination of these two imaging modalities.

To the best of our knowledge, the present study is the first to use a combination of ^19^F-MRI and ^18^F-FDG-PET. Owing to the simplicity of its quantification process and image interpretation, ^19^F-MRI has been suggested as a useful tool for quantitative monitoring of TAMs. The parametric potential of ^19^F-MRI TAM tracking has been suggested in a previous study, which had reported that the ^19^F-MR signal intensity observed in the colon of an inflammatory bowel disease model was correlated with a high chance of developing dysplasia [16]. Unlike SPIO nanoparticles, PFC nanoemulsions do not affect the proton spin of adjacent water molecules; this allows simultaneous measurement of other MR parameters, such as those mentioned above, for further analysis of the tumor microenvironment [20, 32]. Thus, ^19^F-MRI tracking of TAMs in conjunction with ^18^F-FDG-PET is expected to be a valuable bimodal platform that provides complementary information for comprehensive monitoring of the tumor microenvironment.

Yet, several concerns regarding PFC-based TAM tracking remain to be overcome. In most ^19^F-MRI-based TAM-tracking studies, including the present one, TAMs are passively labeled by PFC nanoemulsions, without targeting any specific moieties on the cells. It should be noted that not all TAMs are protumorigenic, and labeling both tumor-promoting and tumor-antagonizing TAMs would compromise the goal of tumor characterization. Therefore, for precise profiling of tumors, the phenotype of fluorinated TAMs should be analyzed, and methods for noninvasive differentiation of tumor-promoting and tumor-antagonizing TAMs should be developed. Another concern is that fluorination of TAMs with PFC nanoemulsions might affect the phenotype and physiology of these cells. In several studies, PFC nanoemulsions have been shown to preserve the original function and differentiation potential of various cell types, including hematopoietic [33, 34], neural [35], and mouse-mesenchymal [36] stem cells. Similar studies should be conducted to investigate the influence of PFC labeling on TAM characteristics. The recently reported intrinsic effects of SPIO nanoparticles on altering TAM polarization also emphasize the need for such investigations [37].

The sensitivity and resolution of ^19^F-MRI should also be improved. In this study, the correlations between ^19^F-MR signal and ^18^F-FDG-PET parameters were only done in tumor-by-tumor basis. To further examine the usefulness of combining ^19^F-MRI and ^18^F-FDG-PET in characterizing tumor heterogeneity, a voxel-by-voxel analysis in a tumor as well as correlation to corresponding histology should be performed. For these analyses to be precise, the spatial resolution of ^19^F-MRI should be improved without compromising the current sensitivity.

## 4. Conclusion

In summary, preliminary results from combining ^19^F-MRI and ^18^F-FDG-PET suggest that ^19^F-MRI tracking of TAMs might aid the characterization of tumors and prediction of tumor development. Comparison of intratumoral distribution of TAMs and the spatial pattern of tumor glucose uptake revealed several degrees of heterogeneity in the tumor microenvironment. A significant positive correlation was observed between ^19^F-MR signal intensity and subsequent tumor growth, while inverse correlations were observed between ^19^F signal intensity and ^18^F-FDG-PET parameters. These results together suggest that ^19^F-MRI tracking of TAMs could potentially be used for tumor characterization and that, in combination with ^18^F-FDG-PET, this method could further expand our understanding of the heterogeneous tumor microenvironment and its impact on tumor prognosis. Since TAMs are becoming popular as significant therapeutic targets for cancer treatment, the combination of ^19^F-MRI and ^18^F-FDG-PET might also serve as a platform for assessment of therapeutic response.

## Figures and Tables

**Table 1:** Correlation of ^19^F signal on day 2 with tumor volume, PET parameters, and their changes over time.

Parameters	*r*	*p*
Δ(day 7 − day 2)		
Tumor volume	0.6242	0.0098
SUV	−0.519	0.0393
SUV_max_	−0.1818	0.5004
% ID/g	−0.510	0.0437

Day 2		
Tumor volume	−0.1684	0.533
SUV	0.155	0.567
SUV_max_	−0.163	0.547
% ID/g	0.1269	0.6396

Day 7		
Tumor volume	0.626	0.0096
SUV	−0.666	0.0049
SUV_max_	−0.663	0.0051
% ID/g	−0.681	0.0037

PET, positron emission tomography; SUV, standardized uptake value; SUV_max_, maximum SUV; % ID/g, percentage injected dose per gram tissue.
